# Characterization of key genes and immune cell infiltration associated with endometriosis through integrating bioinformatics and experimental analyses

**DOI:** 10.1186/s41065-025-00417-4

**Published:** 2025-03-31

**Authors:** Ying Peng, Xiangdong She, Ying Peng

**Affiliations:** Department of Obstetrics and Gynecology, The First Affiliated Hospital of USTC, Division of Life Sciences and Medicine, University of Science and Technology of China, Hefei, Anhui China

**Keywords:** EM, Machine learning, Immune cell infiltration, Molecular markers, Potential drugs

## Abstract

**Backgrounds:**

Endometriosis (EM) is the most common gynecological disease in women of childbearing age. This study aims to identify key genes and screen drugs that may contribute to EM treatment.

**Methods:**

The differentially expressed genes (DEGs) were identified using limma analysis in the GSE11691 dataset. The protein–protein network (PPI) was constructed. Four machine learning methods, including LASSO, SVM-RFE, random forest, and Boruta, were applied to identify the key genes associated with EM. Flow cytometry, wound healing, and migration assays were applied to assess the cell functions of APLNR on hEM15A. The immune cell infiltration of each sample in EM was calculated using a single-sample gene set enrichment analysis (ssGSEA) algorithm. The potential drugs were screened using the Connectivity Map (CMAP) database, based on the DEGs. Finally, the expression levels of the three genes were further validated in the GSE23339 dataset.

**Results:**

One hundred thirty-seven down-regulated genes and 304 up-regulated genes were identified. We identified three key genes associated with EM: APLNR, HLA-DPA1, and AP1S2. The ssGSEA analysis results indicated that these genes play an important role in the development of EM. Moreover, EM immune cell infiltration was tightly associated with these three genes. Finally, several molecular compounds targeting EM were screened with the connectivity map (CMAP) database. ShAPLNR decreased the cell viability of hEM15A, increased the number of apoptotic cells, and significantly decreased the proportion of callus through APLNR in vitro studies.

**Discussion:**

Three genes (APLNR, HLA-DPA1, and AP1S2) may serve as novel therapeutic targets for diagnosing and treating patients with EM.

**Supplementary Information:**

The online version contains supplementary material available at 10.1186/s41065-025-00417-4.

## Introduction

Endometriosis (EM) is the most common gynecological disease in women of childbearing age. It is characterized by endometrial tissues outside the uterine cavity, especially in the pelvic organs [1]. The incidence rate of women of childbearing age is about 10%–15% and 50%–60% of women have pelvic pain and dysmenorrhea. Up to 50% of women have infertility [2]. Due to endometriosis, people suffer from dysmenorrhea, pelvic pain, menstrual disorders, infertility, and dyspareunia [3], with an increased risk of ovarian tumors associated with endometriosis.

Current treatments include drugs and surgery [4], but their efficacy is limited and even affects fertility or causes systemic side effects, such as vasomotor symptoms and osteopenia [5]. In addition, expensive surgery and drug therapy increase social costs [6, 7]. Therefore, endometriosis treatment remains a challenging issue in the medical field.

However, the study of endometriosis has continued for decades; the origin and pathogenesis of ectopic endometriosis lesions are still controversial, with multiple hypotheses that may be related to genetic, immune, and environmental factors [5, 8]. The most widely accepted hypothesis is Sampson's menstrual retrograde theory [9]. Menstrual reflux is a common phenomenon, but only 10% of women develop endometriosis [10].

Cumulative evidence confirms the relationship between the disease and immune factors [11, 12]. Immune system disorders can result in the implantation, multiplication, and angiogenesis of ectopic endometrial tissue [13]. Immune dysfunction in endometriosis patients permits implanting and development of menstrual fragments [13]. Data depicted that the cytotoxic activity of NK cells in the ascites of patients with endometriosis decreased while the number of macrophages and cytokines increased [14]. Phagocytes and NK cells do not target and destroy endometrial cells in the peritoneal cavity. Thus, the mechanism known as "immune escape" can invade the peritoneum [15], leading to disease progression. Guo et al. applied the newly developed multi-parameter single-cell technique and cell counting (CyTOF) to identify and quantify immune cells in the peritoneal fluid and peripheral blood of patients with endometriosis and control. The results demonstrated that both innate and adaptive immune systems play a role in endometriosis [16].

Nonetheless, EM is associated with immune abnormalities, and its role is not understood. In this study, we downloaded the GEO datasets from the GEO database. The key genes were identified using multiple machine learning methods. The expression and diagnostic values were then explored using an external dataset and qPCR experiments. In addition, the relationship between immune cells and key genes was investigated. Several molecular drugs associated with EM were screened using the CMAP drug database.

Overall, the findings of this study may shed light on the molecular mechanisms underpinning EM and aid in identifying potential treatments.

## Materials and methods

### Acquisition and processing of gene expression data

**Fig. 1:**
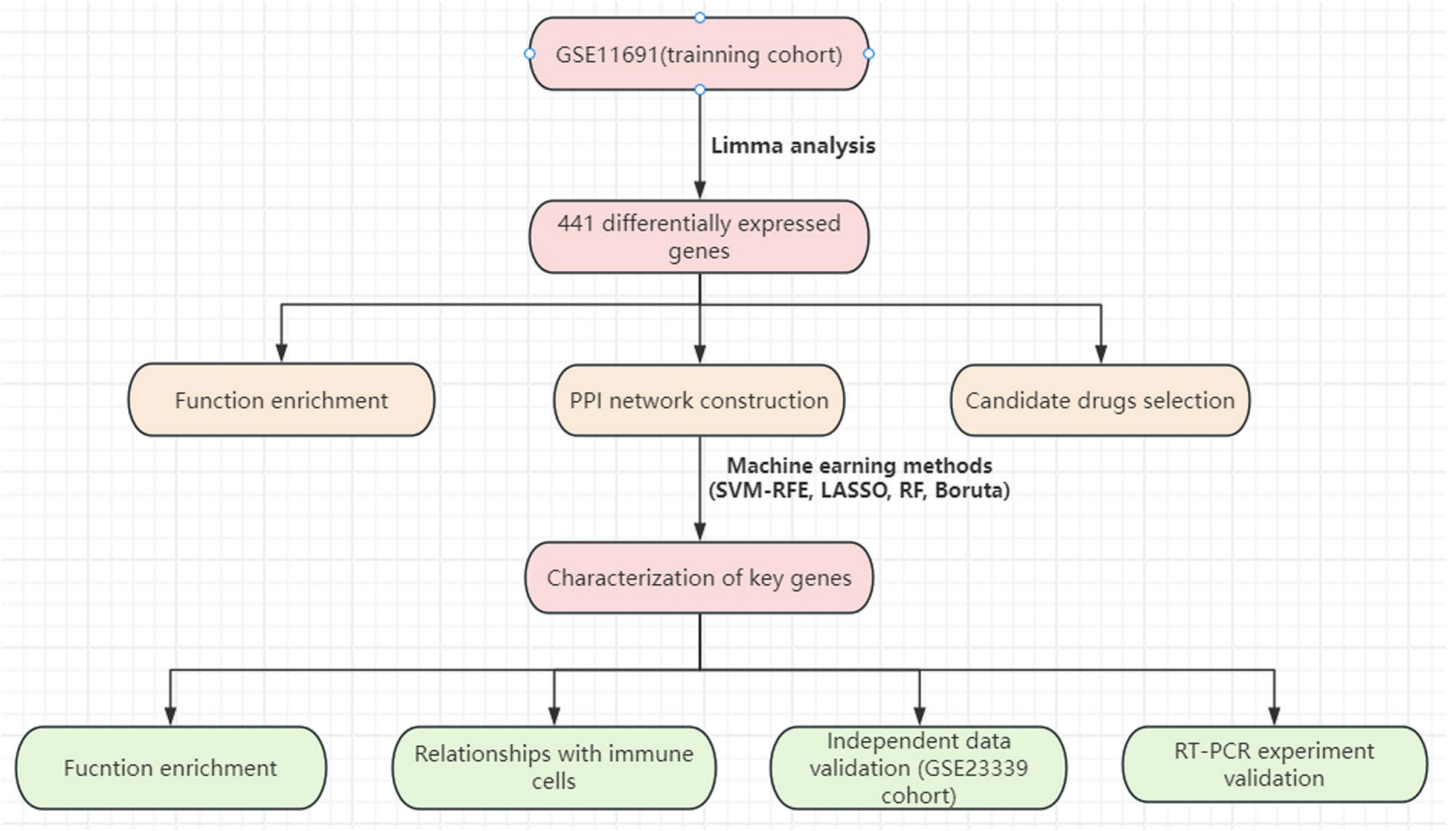
The overall design of the study

We downloaded the raw data of GSE11691 and GSE23339 microarrays (CEL files) from the GEO database. (http://www.ncbi.nlm.nih.gov/geo/). The GSE11691 dataset, which was derived from the GPL96 platform, consists of nine eutopic endometria and nine matched ectopic lesion endometrium [17]. Meanwhile, In the GSE23339 dataset, which was extracted from the GPL6102 platform, ten patients with ovarian endometriosis are matched with nine patients with endometrium samples [18] (Table 1). The detail procedure was showed in Fig. 1.

**Table 1:** The details of cohorts info

Cohorts	GPL platform	Endometrium	Endometriosis
GSE11691	GPL96	9	9
GSE23339	GPL6102	9	10

### Differential expression analysis and enrichment

To identify DEGs, the R package "limma" was used based on the following criteria: FDR < 0.05 and log|FC|> 1 [19]. The "ClusterProfiler" R package analyzed all DEGs for GO and KEGG enrichment [20]. The significant pathways or functions were screened based on an adjusted *p*-value < 0.05.

### Protein–protein interaction network construction

The string database (https://string-db.org/) was used to explore protein–protein interactions. The minimum interaction score for establishing the PPI network was > 0.4, with the disconnected nodes hidden. Cytoscape software was applied to visualize the PPI network. The hub networks were selected using the cytoscape plug-in MCODE software, and top two hub networks genes were screened based on the degree cutoff (degree cutoff:2) and k core (K core: 2) [21].

### Selection of key genes through multiple machine learning methods

Four machine learning algorithms were used to select key genes, including the least absolute shrinkage method and the selection operator (LASSO) [22], SVM-RFE, Boruta, and random forest (RF) algorithms. The LASSO regression was conducted using the "glmnet" package with ten-fold cross validation. The genes associated with 1se.lambda were identified as potential biomarkers. The SVM-RFE algorithm was implemented using the "e1071", "caret", and "kernlab" R packages, with ten-fold cross-validation utilized [23]. The Boruta algorithm was applied to determine the most significant features by comparing the z values of each gene. Additionally, using the “RandomForest” package, the average error rate of candidate genes was calculated to determine the optimal number of variables. The number of trees was chosen based on the lowest error rate, and genes with feature importance scores above 0.3 were selected. After selected the genes from the above four algorithms, we further performed the gene set enrichment analysis (GSEA) to investigate the genes function. The significant pathways were screened based on the *p*-value < 0.05.

### Immune cell infiltration analysis

The immune cell scores were calculated using single-sample gene set enrichment analysis (ssGSEA) based on 28 immune cell gene sets for each sample [24].

### Connectivity Map (CMAP) Drug Analysis

To investigate the potential molecular drugs associated with PE, we uploaded the down-regulated and up-regulated genes to the CMAP drug database (https://clue.io/) [25]. The negative enrichment value of the drugs might have the potential for the treatment of EM. Thus, we screen the drugs with an enrichment score of < − 0.6.

### qRT-PCR analysis

qRT-PCR was carried out using endometrial samples of 20 patients with ovarian endometriosis and 20 with hysteroscopy due to tubal infertility who underwent intrauterine surgery at the Anhui provincial hospital from December 2021 to May 2022. Patients who donated tissues ranged in age from 20 to 45 years. This research has been endorsed by Anhui Provincial hospital ethics committee(NO:2021KY230) and is based on the ethical requirements of the Helsinki Declaration. Real-time PCR was performed with the SYBR Premix Ex Taq (Takara Bio, Beijing, China). β-actin was used as the endogenous control. The relative expression of key genes was calculated using the 2 − ΔΔct method. Primers for each detection index are listed in Table 1.

### Statistics

Data were analyzed by GraphPad Prism (version 9.1.1.225, GraphPad Software Inc.). The data were presented as mean ± SD. Student t-test was performed for the two-group comparison, and analysis of variance (ANOVA) was used for the comparison among multiple groups followed by Duncan’s post-hoc test. The "pROC" R package was used to perform ROC analysis. The correlation analysis was performed using the spearman correlation coefficient. *P* < 0.05 was deemed as significant difference.

## Results

### DEG Identification and PPI network construction

**Fig. 2:**
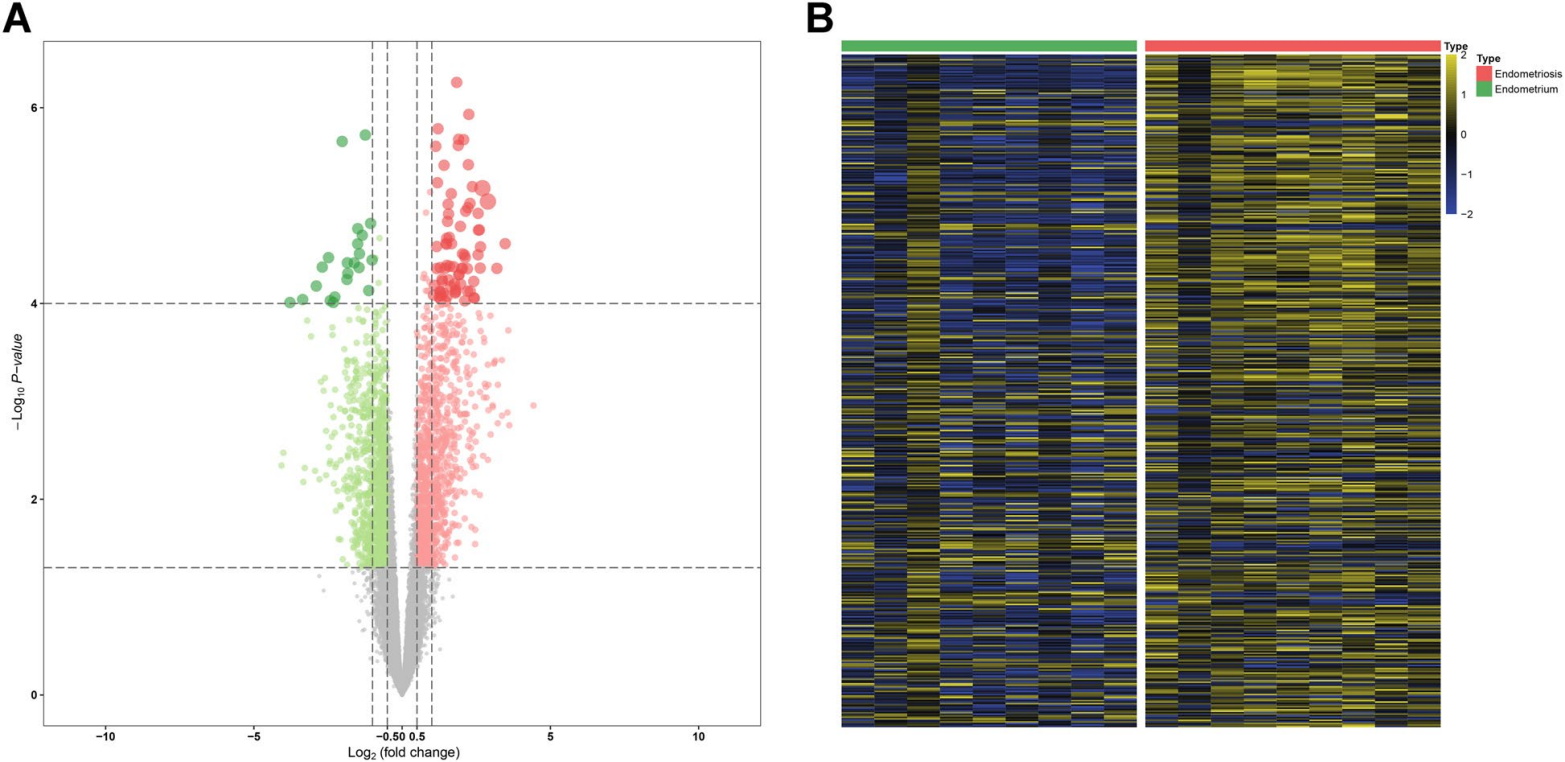
Identification of differentially expressed genes in GSE11691. **A** Volcano plot of the genes, the green dots represent the down-regulated genes, red dots represent the up-regulated genes, while the black dots showed genes with no significance. **B** A heatmap plot of the differentially expressed genes

**Fig. 3:**
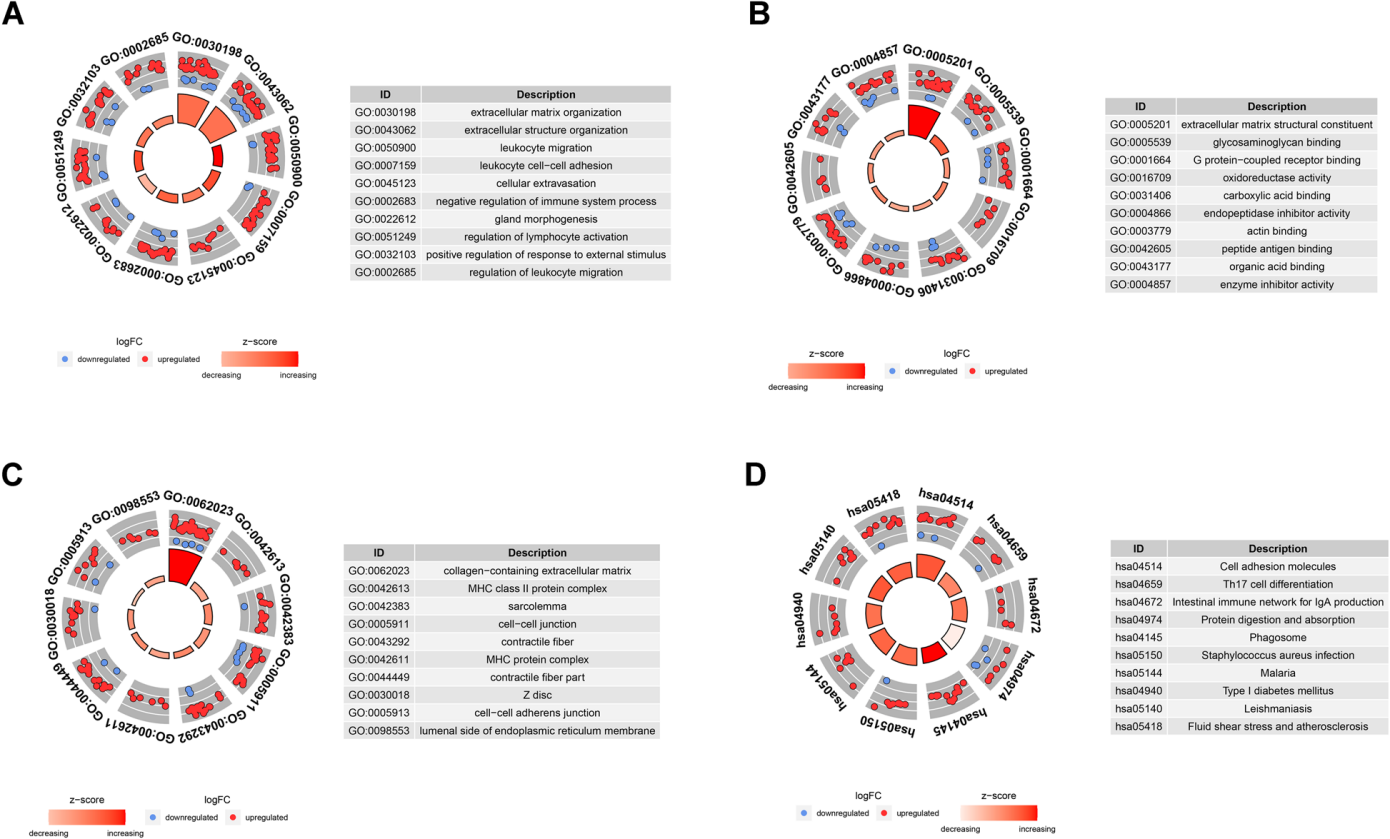
Gene ontology and KEGG enrichment analysis results of the DEGs. **A**-**C** GO enrichment analysis including BP, MF, CC; (D) KEGG pathway enrichment analysis

Among the GSE11691 dataset, there were nine endometrium and nine endometriosis samples, respectively. Through the DEG analysis between the two groups, we identified 137 down-regulated genes and 304 up-regulated genes separately (Fig. 2A-B) (Supplementary TableS1). We then performed a GO enrichment analysis based on the 441 DEGs. The biological process (BP) analysis revealed that genes were enriched in an extracellular matrix organization, leukocyte migration, cell–cell adhesion, and regulation of lymphocyte activation (Fig. 3A). The molecular function (MF) enrichment analysis revealed that genes are mainly involved in glycosaminoglycan binding, carboxylic acid binding, endopeptidase inhibitor activity, and actin-binding (Fig. 3B). The cellular component (CC) demonstrated that genes were enriched in the MHC class II protein complex, cell − cell junction, and MHC protein complex (Fig. 3C).

**Fig. 4:**
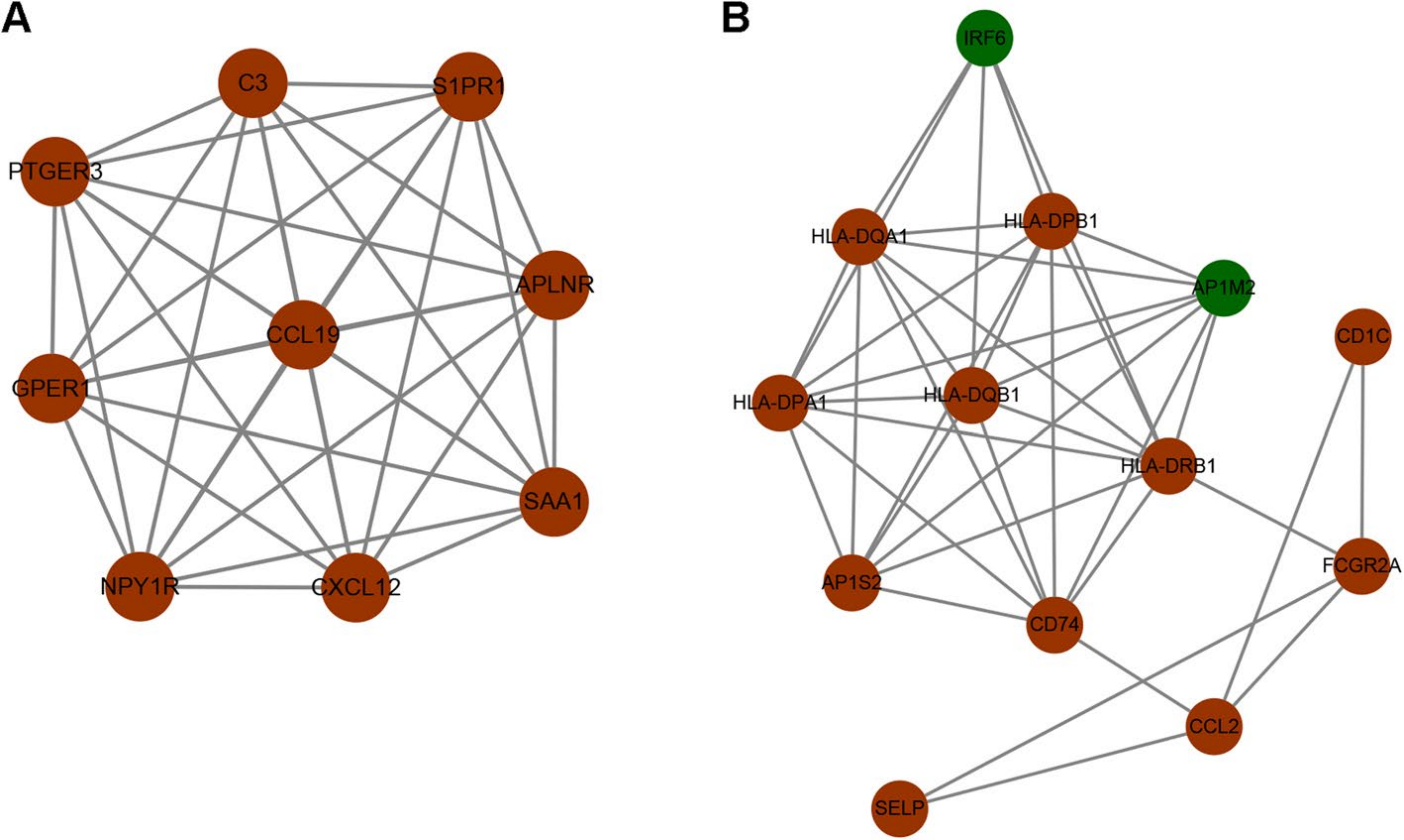
The protein interaction network diagram. **A** The protein interaction diagram of all differential genes. **B** Two hub network screened by the MCODE algorithm

KEGG pathway analysis was also conducted on these DEGs; the top terms are shown in Fig. 3D. The enriched KEGG terms revealed that these DEGs are mainly involved in cell adhesion molecules, Th17 cell differentiation, protein digestion and absorption, and Phagosome. In addition, a protein–protein interaction (PPI) network was constructed using these DEGs, and the MCODE algorithm was used to screen the top two hub networks (Fig. 4A-B). These hub networks included 22 hub genes (20 up-regulated and two down-regulated genes).

### Key gene selection and validation

**Fig. 5:**
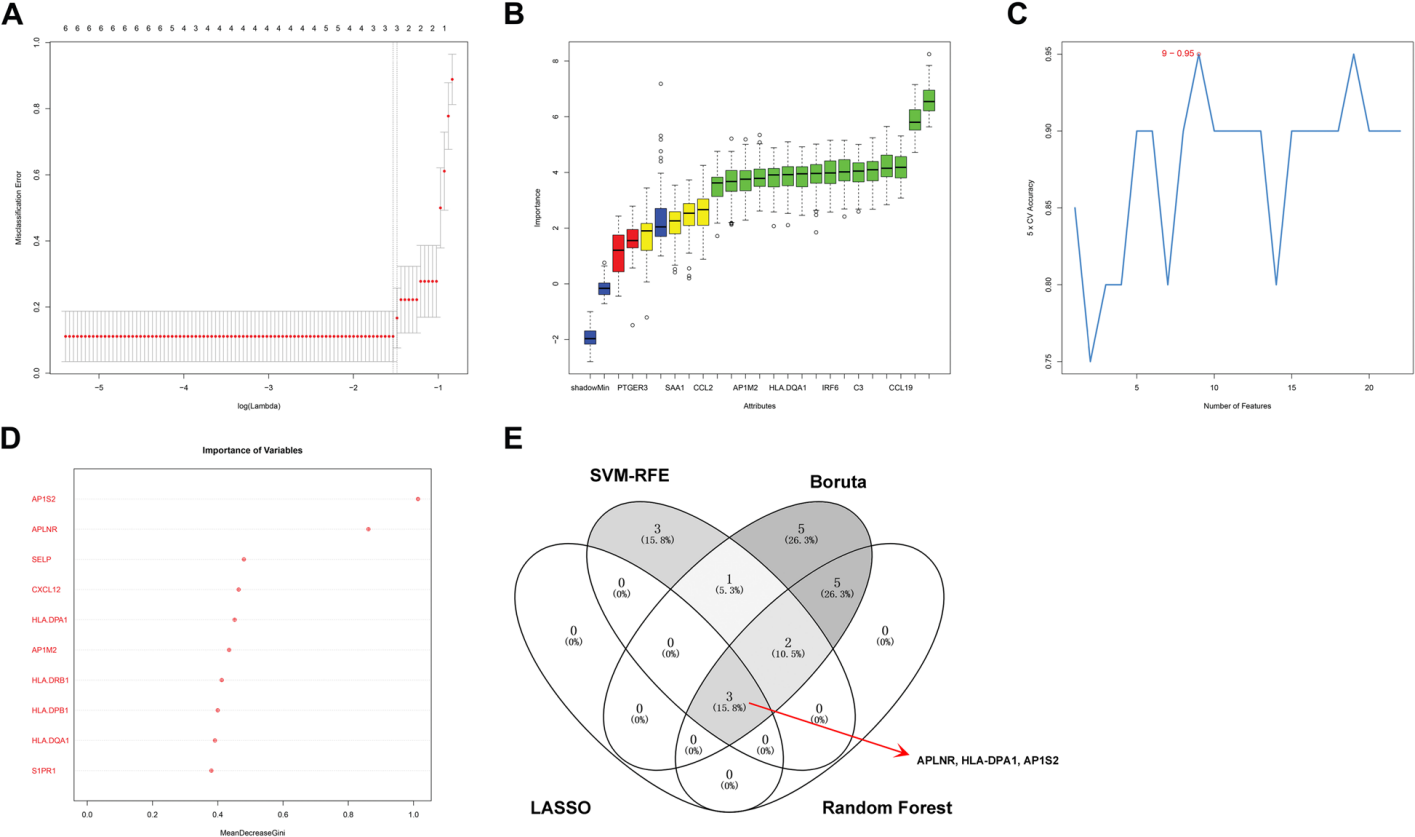
Identification of diagnostic markers. **A** Three genes including APLNR, HLA-DPA1 and AP1S2 were obtained using the least absolute shrinkage and selection operator [27]. **B** Sixteen genes corresponding to the EM were obtained by the boruta algorithm. **C** Support vector machine-recursive feature elimination (SVM-RFE) algorithm to screen diagnostic markers. **D** Ten genes were defined as key genes by random forest. **E** Venn diagram showing the intersection among diagnostic markers between the four algorithms

According to the 22 hub genes, we further applied four machine learning methods to screen key regulators associated with EM. First, we chose a random seed and found that the number of variables with the smallest score deviation was three. Therefore, three genes, including APLNR, HLA-DPA1, and AP1S2, were obtained from the lasso analysis results (Fig. 5A). The Boruta algorithm also set the same random seeds and obtained 16 genes corresponding to the EM (CCL19, NPY1R, APLNR, CXCL12, S1PR1, C3, CD74, HLA-DQB1, HLA-DQA1, HLA-DRB1, HLA-DPA1, HLA-DPB1, IRF6, AP1M2, AP1S2, and SELP) (Fig. 5B). Using SVM-RFE for tenfold validation, the minimum root means square error [26] was identified with nine genes, including CD1C, APLNR, AP1S2, HLA-DPA1, AP1M2, SELP, SAA1, CCL19, and GPER1 (Fig. 5C). In addition, we applied the RF analysis to the EM; the genes were ranked according to their relative importance, and the ten most important genes were defined as key genes (Fig. 5D). Finally, we integrated the four machine learning method results and identified three key genes: APLNR, HLA-DPA1, and AP1S2 (Fig. 5E).

### Gene function enrichment

**Fig. 6:**
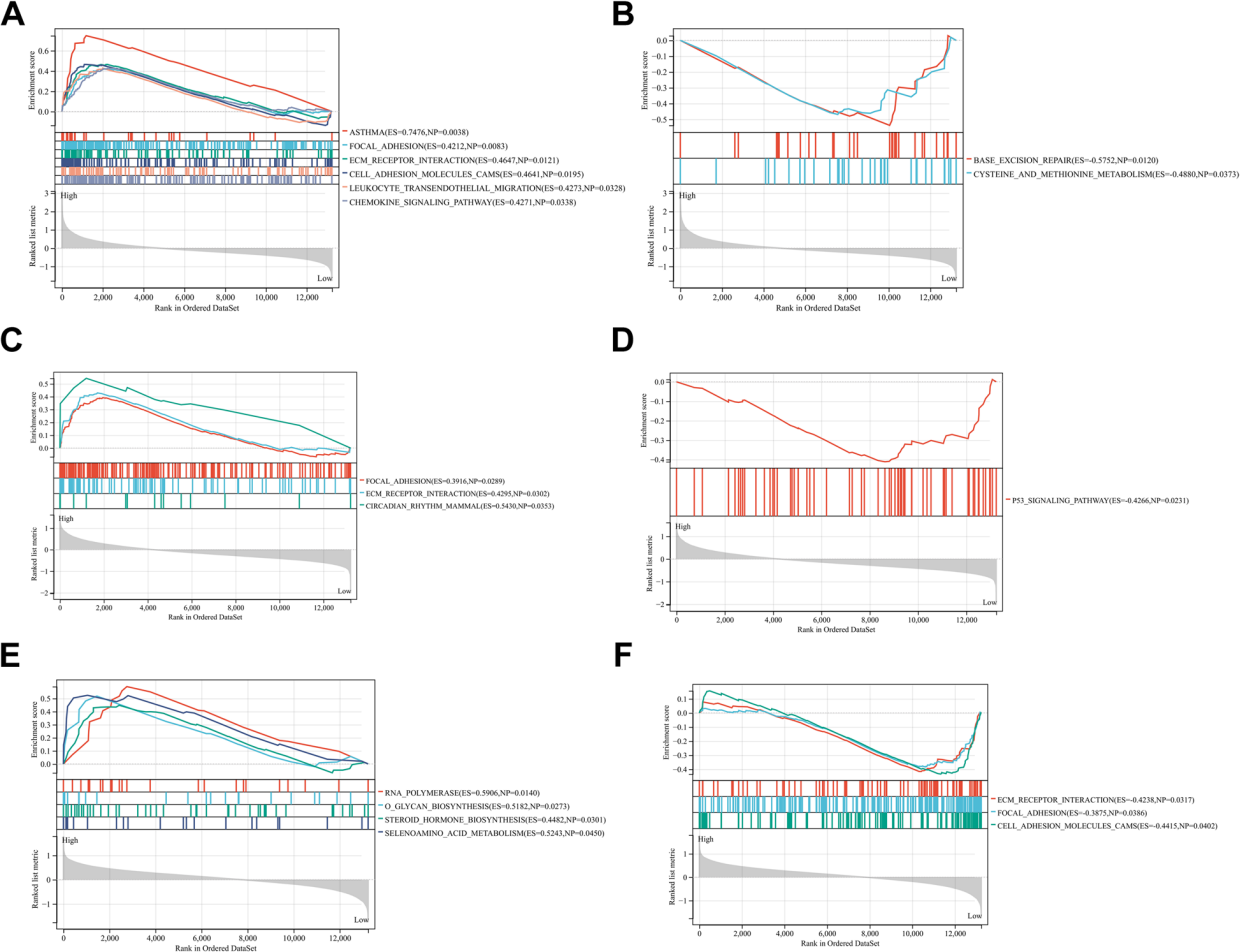
Gene set enrichment analysis (GSEA) was performed for the three key genes based on the median expression profile. **A**,**B** The significant pathways were enriched in the high and low expression group of AP1S2, respectively. **C**,**D** The significant pathways were enriched in the high and low expression group of APLNR, respectively. **D**,**E** The significant pathways were enriched in the high and low expression group of HLA-DPA1, respectively

To further explore the gene MF, we performed a GSEA enrichment analysis on these genes. According to the median expression level of the three genes, we categorized the samples into high- and low-expression groups, respectively. The high-expression group of AP1S2 was significantly enriched in ECM receptor interaction, cell adhesion molecular cams, chemokine signaling pathway, and focal adhesion. The low-expression group of AP1S2 was involved in base exclusion repair and cysteine and methionine metabolism (Fig. 6A-B). In the APLNR, ECM receptor interaction and focal adhesion were enriched in the high-expression group, while the p53 signaling pathway was enriched in the low-expression group (Fig. 6C-D). In addition, we observed that RNA polymerase and O glycan biosynthesis were mainly enriched in the high-expression group of HLA-DPA1, while ECM receptor interaction, focal adhesion, and cell adhesion molecular cams were enriched in the low-expression group of HLA-DPA1 (Fig. 6E-F).

### Immune enrichment analysis

**Fig. 7:**
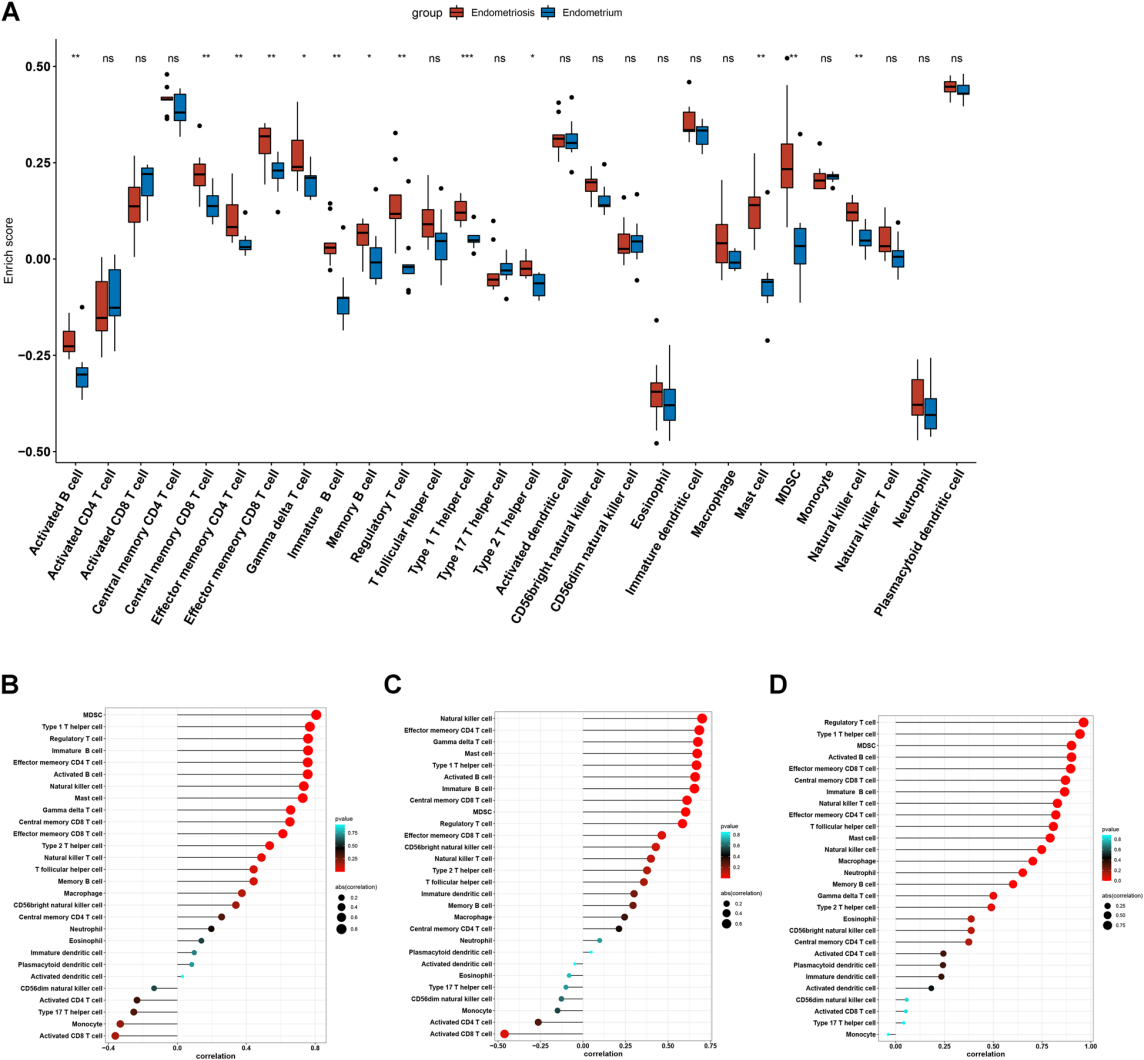
Immune enrichment analysis. **A** Profile of the level of 22 immune cells’ infiltration in the EM and normal tissues. **B** Correlation between AP2S1 and 22 immune cells. **C** Correlation between APLNR and 22 immune cells. **D** Correlation between HLA-DPA1 and 22 immune cells.The size of the dots represents the strength of the correlation between genes and immune cells; with larger dots implying a stronger correlation, and vice versa. The color of the dots represents the *p*-value

**Fig. 8:**
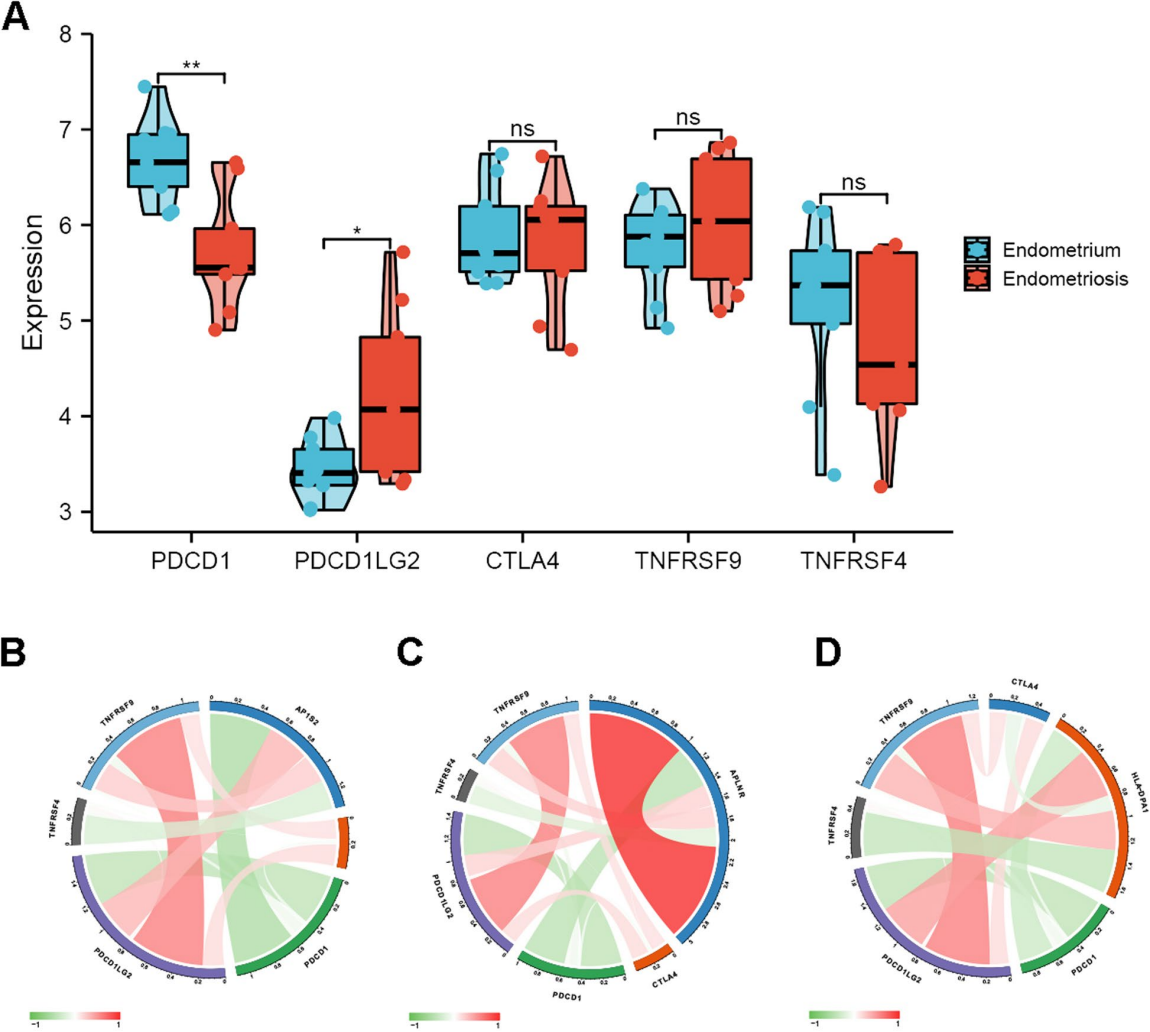
The immune checkpoint analysis in EM. **A** Evaluation of immune checkpoint genes the between endometriosis and endometrium. Correlation analysis between immune checkpoint genes, AP1S2 (**B)**, APLNR (**C**) and HLA-DPA1 (**D**)

The immune cell plays a vital role in EM, and we thus investigated the role of the immune cell between endometriosis and endometrium. Interestingly, most immune cell enrichment levels in endometriosis were significantly higher than in the endometrium (Fig. 7A), including activated B cells, central memory CD8 T cells, effector memory CD4 T cells, effector memory CD8 T cells, gamma delta T cells, immature B cells, memory B cells, regulatory T cells, type 1 T helper cells, type 2 T helper cells, mast cells, and MDSC. We then explored the correlation between the three key genes and immune cells. The AP1S2 positively correlated with MDSC and negatively with an activated CD8 T cell (Fig. 7B). APLNR was positively related to natural killer cells and negatively related to activated CD8 T cells (Fig. 7C). HLA-DPA1 was positively associated with regulatory T cells (Fig. 7D). In addition, we explored the immune checkpoints (PDCD1, PDCD1LG2, CTLA4, TNFRSF9, and TNFRSF4) expression levels between the endometrium and endometriosis. We found that PDCD1 was significantly higher in the endometrium, while PDCD1LG2 was substantially higher in endometriosis (Fig. 8A). Immune checkpoints and key genes were also examined; we found that AP2S1, APLNR, and HLA-DPA1 were negatively correlated with TNFRSF4 and PDCD1, while they were positively correlated with PDCD1LG2 and TNFRSF9 (Fig. 8B-D).

### Characterization of potential drugs for the treatment of EM

**Fig. 9:**
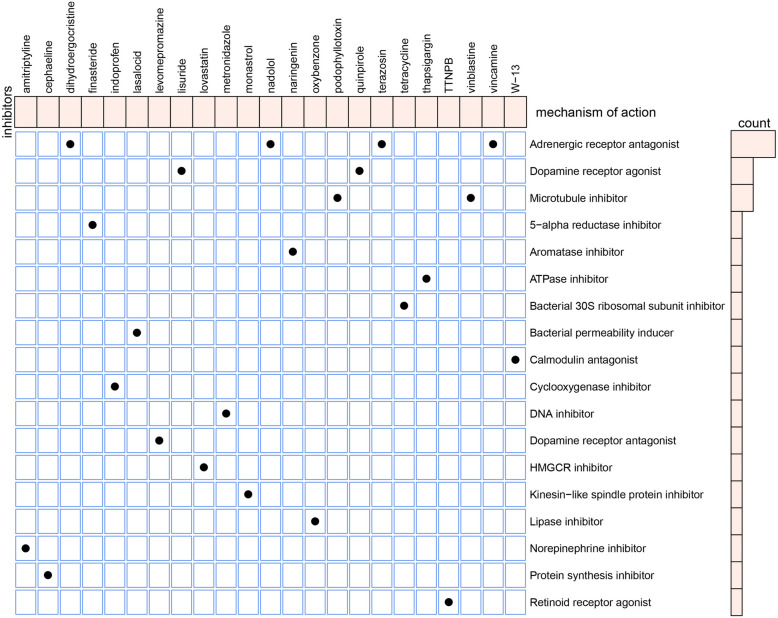
Identification of the small molecular compounds and its mechanism of action for the treatment of EM

According to the 441 DEGs, we uploaded the down-regulated and up-regulated genes to the connectivity map (CMAP) drug database. We then downloaded the drug enrichment results from the database and screened 23 molecular drugs with molecular of action (MOA) (Fig. 9). Among these drugs, dihydroergocristine, nadolol, terazosin, and vincamine shared the MOA of adrenergic receptor antagonists. Lisuride and quinpirole shared the MOA of dopamine receptor agonists. Thapsigargin shared the MOA of the ATPase inhibitor. Metronidazole shared the MOA of the DNA inhibitor.

### Validation of key genes

**Fig. 10:**
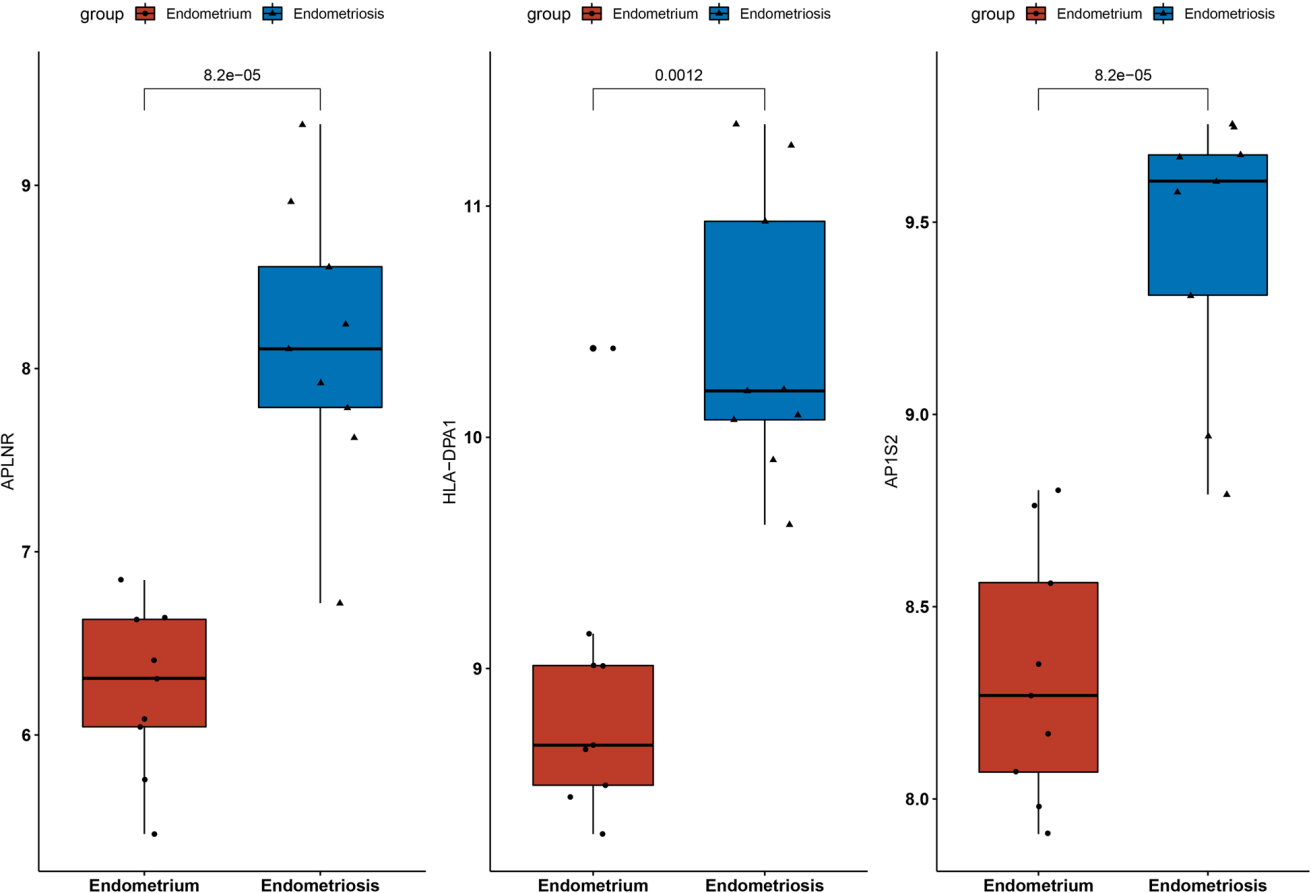
Expression levels of three key genes in GSE11691 between EM and normal tissues, **A** AP2S1, **B** APLNR, **C** HLA-DPA1

**Fig. 11:**
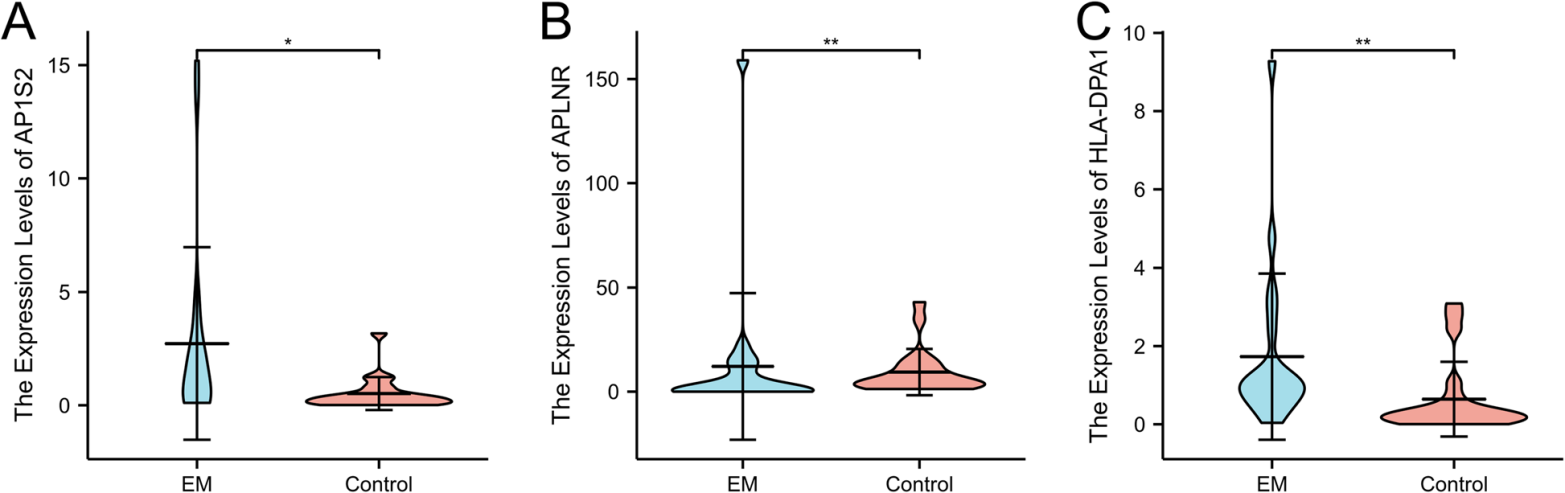
The relative expression of 3 key genes between EM and normal tissues using RT-PCR experiment. * *P* < 0.05; **P < 0.01. **A** AP2S1, **B** APLNR, **C** HLA-DPA1

We investigated the expression levels of the three genes between the endometrium and endometriosis in the GSE11691 cohort and found that all genes presented a high-expression level in endometriosis (Fig. 10). The ROC curve analysis result indicated that the three genes have a good diagnostic value for EM (0.926–0.988) (Figure S1). We then validated the expression levels of the three genes in the external cohort GSE23339. HLA-DPA1 presented a significantly higher level in EM (Figure S2), which is consistent with our results. Moreover, the ROC curve analysis also revealed good performance for the EM (0.667–0.811) (Figure S3). Lastly, we used the RT-PCR experiment to validate the expression level of the three genes, and we found that APLNR, HLA-DPA1, and AP1S2 expression levels were significantly up-regulated in the EM tissue compared to normal tissue, which is consistent with the results of the bioinformatics analysis (Fig. 11).

## Discussion

In the present study, we identified 441 DEGs in the GSE11691 dataset between endometrium and endometriosis tissues. These DEGs included 233 up-regulated and 190 down-regulated genes, respectively. GO analyses revealed that these genes were enriched in the positive regulation of extracellular matrix structural constituents, extracellular matrix organization, and collagen − containing extracellular matrix. KEGG enrichment analysis revealed that these DEGs were involved in the cell adhesion molecules signaling pathway, Th17 cell differentiation signaling pathway, and intestinal immune network for IgA production signaling pathway. Our results prompted the hypothesis that these enriched functions and pathways might have immune effects on EM. Moreover, three key genes, APLNR, HLA-DPA1, and AP1S2, were identified by integrating the LASSO, RF, SVM-RFE, and Boruta algorithms.

The APLNR is a 380 amino acid-residue transmembrane AG protein-coupled receptor [28], also known as APJ. Apelin is an endogenous ligand for APJ. Hearts, lungs, livers, brains, limbs, skin, kidneys, retinas, and adipose tissues express apelin and its receptor APJ [29], and they are involved in a wide range of physiological functions. The apelin/APJ system is closely related to the occurrence and development of tumors, such as cholangiocarcinoma [30], osteosarcoma [31], glioblastoma [32], and renal cell carcinoma [33]. The dysregulation of APLNR in ovarian clear cell carcinoma (OCCC) cells was associated with growth, migration, and cell cycle progression (Xu and Shen, 2018). Various studies have shown that APLN (apelin) regulates female and male reproduction as part of the apelinergic system found in the hypothalamus, pituitary, and gonadotrophin axis. [34]. Analyses of developmental model organisms suggested an important role for the apelin/APJ system in embryonic angiogenesis (Kidoya et al., 2008). However, the role of APLNR in EM remains unclear.

The HLA-DPA1 antigen receptor is an analog of the HLA Class II chain and participates in the immune response and the presentation of antigenic peptides as an MHC class II antigen receptor. [35]. Lower expression of HLA-DPA1 expression is associated with a poor prognosis in patients with multiple myeloma and adrenal cortical tumors [36, 37]. Homozygous RS1431403 genotypes (CC and TT) may increase the risk of non-fatal pregnancy through the abnormal increase of HLA-DPA1 levels [38]. Infertile patients with or without endometriosis demonstrated reduced HLA-DPA1 and HLA-DPB1 expression in their endometriums [39].

AP1S2 is a protein encoded by the AP1S2 gene in the human body, which is located on Xp22.2 and is responsible for recognizing transmembrane receptor sorting signals and clathrin recruitment [40]. According to many studies, AP1S2 mutations are associated with mental retardation [41, 42], which is significant for genetic counseling and prenatal diagnosis. However, the role of AP1S2 in EM remains unclear.

This study identified distinct small molecules associated with EM-based on DEGs using the CAMP database. Several drugs, including finasteride, indoprofen, levomepromazine, lisuride, metronidazole, thapsigargin, TTNPB, and vinblastine, demonstrated a high negative enrichment value. Finasteride is a competitive inhibitor of human 5α reductase type II (5α R2) that blocks the conversion of testosterone to dihydrotestosterone (DHT) in the outer epithelial sheath and the dermal papilla [43]. Indoprofen is a nonsteroidal anti-inflammatory drug with minimal side effects [44] that improves muscle strength and muscle mass in elderly mice and muscular atrophy models. It can also serve as a chemical probe to identify proteins that regulate SMN protein production [45]. Furthermore, metronidazole is a synthetic antigenic animal and antimicrobial agent belonging to nitroimidazoles that can treat various infectious diseases. In one study on the relationship between metronidazole and endometriosis, the volume of ectopic lesions in mice who were given metronidazole was significantly smaller than that in the control group (P < 0.001). In addition, metronidazole antibiotic treatment could reduce the progression of endometriosis in mice [46]. Accumulative research is needed to further explore the relationship between metronidazole and endometriosis. Vinblastine is a microtubule polymerization inhibitor, a well-known anticancer drug that can effectively treat many types of tumors, such as Hodgkin's disease, lymphocytic lymphoma, advanced breast cancer, and choriocarcinoma [47, 48]. Pre-clinical studies have shown that in addition to its direct antitumor cytotoxicity, levomepromazine, a lipoid antipsychotic drug containing the formula C19H25N2OS phenothiazine, can be used to relieve bronchoconstriction [49], preoperative sedation [50], end-stage and postoperative analgesia [51], and control of nausea and vomiting [52]. In folk medicine, thapsigargin treats rheumatoid arthritis, lung diseases, and female infertility. Thapsigargin is a phytochemical found in the roots and fruits of Mediterranean plants from the *Thapsia L*. species that has been used for centuries in folk medicine to treat rheumatic pain and lung diseases [53]. Thapsigargin has been found effective cytotoxin that induces endoplasmic reticulum stress and apoptosis [54, 55], opening up its prospects as an anticancer agent. TTNPB, an analog of all-trans retinoic acid, regulates cell growth and differentiation, and its teratogenicity is nearly three orders of magnitude higher than that of atRA, which limits the development of TTNPB in humans [56]. TTNPB is used for neural differentiation [57] and can activate all retinoic acid receptors. Lisuride, a semisynthetic ergot derivative, was first used clinically to treat migraine headaches (Zikan and Siemonsky, 1960). Several studies have reported that lisuride stimulates post-synaptic dopamine receptors (Rosenfeld and Makman 1981; Uzumaki et al. 1982; Schechter 1984; Cunningham et al. 1987a). Transdermal administration of lisuride can improve the frequency and intensity of movement fluctuations in patients with Parkinson's disease [58]. Zweckberger et al. studied ergoethyluride in rats with controlled cortical impact injury, and it seemed to have a significant anticonvulsant effect [59]. Endometriosis drug development still faces significant transformation challenges, and more pre-clinical studies are needed.

In conclusion, we identified three potential diagnostic biomarkers and therapeutic target genes (APLNR, HLA-DPA1, and AP1S2) associated with EM. In addition, the potential therapeutic drug for endometriosis treatment was analyzed as finasteride, indoprofen, levomepromazine, lisuride, metronidazole, thapsigargin, TTNPB, and vinblastine. This paper presented novel insights into EM at the immunological and molecular levels, but further research is needed to validate our findings.

### Limitations

This paper presented novel insights into EM at the immunological and molecular levels, but there are some limitations. Though the dependability of the original microarray was conducted and validated by RT-PCR, the results are constrained due to the small sample size. Second, despite the identification of three key genes as prospective biomarkers for EM immunotyping, more research on the functions and regulatory mechanisms of key genes in EM is still needed. As a result, this will be the focus of future efforts.

## Supplementary Information

Supplementary Material 1.

Supplementary Material 2.

Supplementary Material 3.

Supplementary Material 4.

## Data Availability

No datasets were generated or analysed during the current study.
