# Supplementary figures and images for: Characterization of key genes and immune cell infiltration associated with endometriosis through integrating bioinformatics and experimental analyses
Source: Hereditas. 2025 Mar 31;162:49. doi: 10.1186/s41065-025-00417-4 (PMC11956255; doi:10.1186/s41065-025-00417-4)

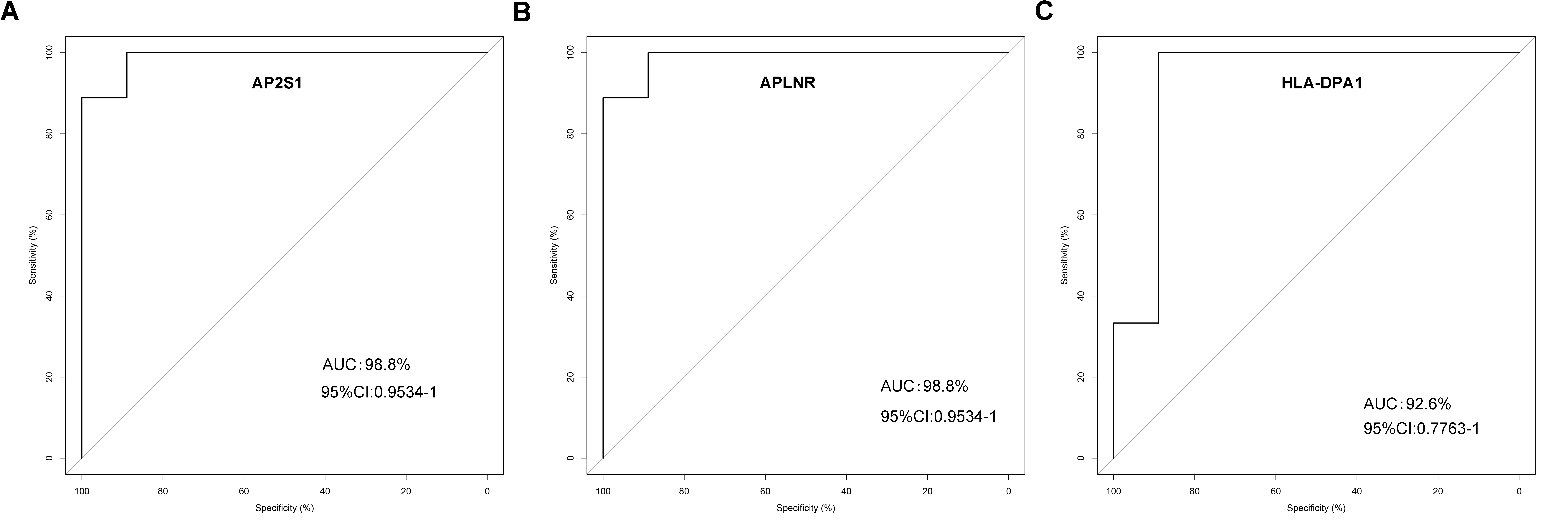

Supplement: Supplementary file 1 — Supplementary Material 1. [file 41065_2025_417_MOESM1_ESM.tif]

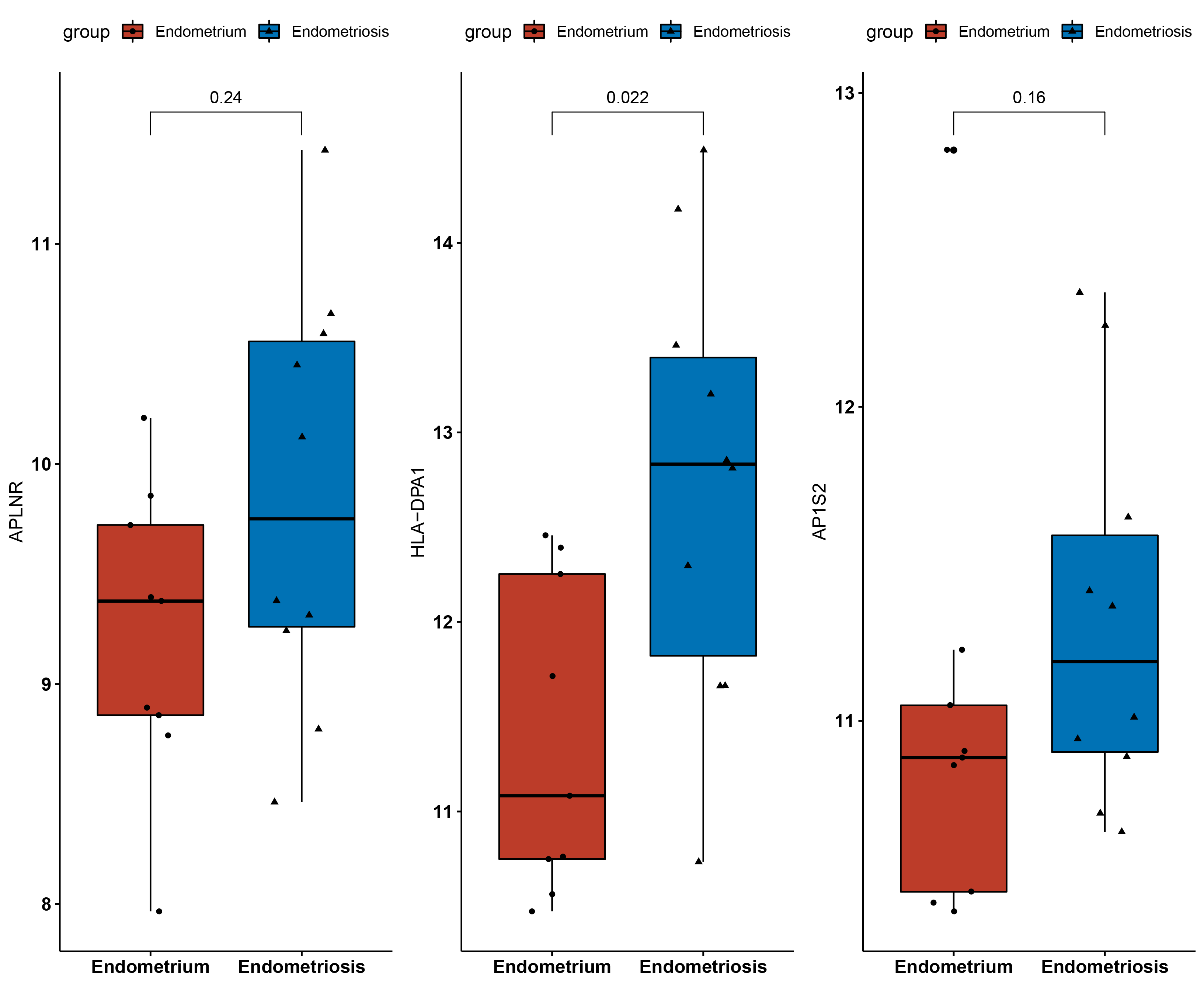

Supplement: Supplementary file 2 — Supplementary Material 2. [file 41065_2025_417_MOESM2_ESM.tif]

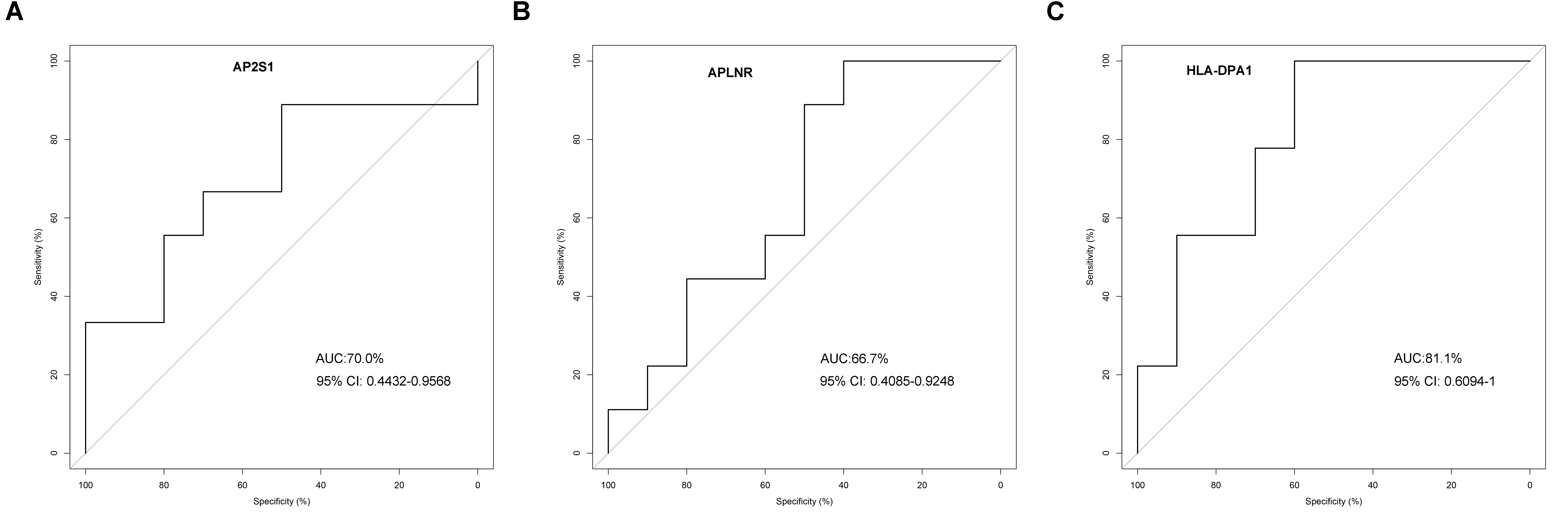

Supplement: Supplementary file 3 — Supplementary Material 3. [file 41065_2025_417_MOESM3_ESM.tif]
